# Realist explanatory theory building method for social epidemiology: a protocol for a mixed method multilevel study of neighbourhood context and postnatal depression

**DOI:** 10.1186/2193-1801-3-12

**Published:** 2014-01-05

**Authors:** John G Eastwood, Bin B Jalaludin, Lynn A Kemp

**Affiliations:** South Western Sydney Local Health District, Locked Mail Bag 7279, Liverpool BC 1871, Sydney, NSW Australia; School of Public Health and Community Medicine, The University of New South Wales, Sydney, NSW 2052 Australia; School of Women’s and Children’s Health, The University of New South Wales, Sydney, NSW 2052 Australia; School of Public Health, University of Sydney, Sydney, NSW 2006 Australia; School of Public Health, Griffith University, Gold Coast, QLD 4222 Australia

## Abstract

A recent criticism of social epidemiological studies, and multi-level studies in particular has been a paucity of theory. We will present here the protocol for a study that aims to build a theory of the social epidemiology of maternal depression. We use a critical realist approach which is trans-disciplinary, encompassing both quantitative and qualitative traditions, and that assumes both ontological and hierarchical stratification of reality. We describe a critical realist *Explanatory Theory Building Method* comprising of an: 1) emergent phase, 2) construction phase, and 3) confirmatory phase. A concurrent triangulated mixed method multilevel cross-sectional study design is described. The *Emergent Phase* uses: interviews, focus groups, exploratory data analysis, exploratory factor analysis, regression, and multilevel Bayesian spatial data analysis to detect and describe phenomena. Abductive and retroductive reasoning will be applied to: categorical principal component analysis, exploratory factor analysis, regression, coding of concepts and categories, constant comparative analysis, drawing of conceptual networks, and situational analysis to generate theoretical concepts. The *Theory Construction Phase* will include: 1) defining stratified levels; 2) analytic resolution; 3) abductive reasoning; 4) comparative analysis (triangulation); 5) retroduction; 6) postulate and proposition development; 7) comparison and assessment of theories; and 8) conceptual frameworks and model development. The strength of the critical realist methodology described is the extent to which this paradigm is able to support the epistemological, ontological, axiological, methodological and rhetorical positions of both quantitative and qualitative research in the field of social epidemiology. The extensive multilevel Bayesian studies, intensive qualitative studies, latent variable theory, abductive triangulation, and *Inference to Best Explanation* provide a strong foundation for *Theory Construction*. The study will contribute to defining the role that realism and mixed methods can play in explaining the social determinants and developmental origins of health and disease.

## Background

### Introduction

We have previously reported on individual level psychosocial predictors of postnatal depression in South Western Sydney (Eastwood et al. 2011) and proposed that the findings were consistent with group-level socioeconomic deprivation, neighbourhood environment, social networks and ethnic diversity having causal effects on postnatal depressive symptomatology and other perinatal outcomes. That proposition was consistent with a recent qualitative study of pathways from neighbourhoods to mental well-being which found that neighbourhood affordability, negative community factors including crime and vandalism, and social makeup including unemployment and poverty, were felt to be associated with poor mental well-being (O'Campo et al. 2009b).

A recent criticism of social epidemiological studies, and multi-level studies in particular has been a paucity of theory (Muntaner 1999; Krieger 2001; O'Campo 2003; Carpiano and Daley 2006; Raphael 2006). Muntaner called for social epidemiologists to abandon the Humean notion of causality and adopt “a realist philosophy that favours generating social theory in addition to observation” (Muntaner 1999, p 124).

We will describe here the protocol for a critical realist mixed method and multilevel study that aims to build a theory of the social epidemiology of maternal depression by exploring the individual and ecological-level causal concepts related to postnatal depression. We contend that critical realism, as articulated by Bhaskar (1975) is an appropriate meta-theory for the generation of causal explanations in social epidemiology and may provide answers to the criticisms put forward by Muntaner (1999), Krieger (2001), O'Campo (2003), Carpiano and Daley (2006) and Raphael (2006). The critical realist approach used here assumes both ontological and hierarchical stratification of reality (Danermark 2002) making it suitable for the examination of social phenomenon such as socio-economic stratification, social networks and support, discrimination, work demands and control.

As theory building involves inductive, abductive, retroductive and deductive processes we will use a critical realist approach which is trans-disciplinary, encompassing both quantitative and qualitative traditions, and which assumed both ontological and hierarchical stratification of reality (Danermark 2002). The proposed study will contribute to methodological approaches to theory building, perinatal and infant social epidemiology and our theoretical understanding of how economic, social, physical and political factors might influence developmental and life-course outcomes.

### Critical realism

Realism has been defined as “the view that entities exist independently of being perceived or independently of our theories about them” (Phillips 1987, p 205). Based primarily on the writings of Bhaskar (1975,1998) contemporary critical realism is an ontologically-based philosophy of science that attempts to answer the question ‘what must reality be like to make science possible?’ (Bhaskar 1975). A key feature of critical realism is what Bhaskar refers to as the ‘epistemic fallacy’; by which he means the tendency to couple ontology and epistemology and to confuse that which exists with the knowledge we have about it (what we believe).

The term critical realism was not initially used by Bhaskar who used ‘Transcendental Realism’ in *A Realist Theory of Science* (Bhaskar 1975) to argue that scientific theories were best understood as provisional statements about the characteristics of entities that exist in the natural world. Bhaskar later extended his writing into the social sciences using the term ‘Critical Naturalism’ in *The Possibility of Naturalism* (Bhaskar 1978) which sought to show that social structures exist and that it is possible to study them in the same way as natural ones. Thus Bhaskar has described a philosophy of science that will enable us examine and explain social structures impacting on the health of populations.

A central aspect of critical realism ontology is the distinction between three ontological domains: the empirical, the actual and the real. The empirical domain comprises of our experiences of what actually happens (i.e. experiences) and the actual is constituted by the things that happened independently of whether we observed them or not (i.e. events). The last ontological domain is the deepest level of reality and is constituted by mechanisms with generative power (Jeppesen 2005). A second critical realist ontological dimension is that reality is stratified. Reality is assumed to consist of hierarchically ordered levels where a lower level creates the conditions for a higher level. Each stratum is separate and distinct and may interact with the layer above or below to produce new mechanisms, objects and events. It is the existence of such level-specific mechanisms that constitute a level. The ability of mechanisms to combine to create something new is called *emergence* (McGuire 2006; Danermark 2002; Bhaskar 1998). The concept of mechanisms is central to critical realist ontology. The mechanisms can exist beneath the empirical surface and are not directly observable. Based on observed phenomena the task may be to find the underlying mechanisms that produce the phenomena and to “understand the interplay between them and how they shape the outcome” (Danermark 2002, p 59). C*ontext* thus determines how a mechanism is empirically manifest.

Sayer (2000, p 13) notes that “one of the distinctive features of realism is its analysis of causation which rejects the standard Humean “sucessionist” view that it involves regularities among sequences of events”. As discussed above the realist interpretation makes a distinction between the real and actual with “generative” or causal powers that may, or may not, be activated depending upon other conditions (context, other mechanisms). This is particularly important in the field of social epidemiology where social processes are typically dependent upon the actions of “actors” in the various social and organisational structures. Thus for realists causation is not understood based on a model of regular succession of events. “What causes something to happen has nothing to do with the number of times we observe it happening. Explanation depends instead on identifying causal mechanism and how they work, and discovering if they have been activated and under what conditions” (Sayer 2000, p 14).

Causal inference is the process of drawing conclusions regarding causation by applying forms of reasoning or logic. Danermark et al. (2002, p 79) define inference as “a way of reasoning towards an answer to questions such as: What does this mean? What follows from this? What must exist for this to be possible?” Realists distinguish between four modes of inference: deduction, induction, abduction and retroduction. These forms of reasoning are defined as follows:

*Deduction:* To derive logically valid conclusions from given premises. To derive knowledge of individual phenomena from universal laws.

*Induction:* From a number of observations to draw universally valid conclusion about a whole population. To see similarities in a number of observations, and draw the conclusion that these similarities, also apply to non-studied cases. From observed co-variates to draw conclusions about law-like relations.

*Abduction:* To interpret and recontextualise individual phenomena within a conceptual framework or a set of ideas. To be able to understand something in a new way by observing and interpreting this something in a new conceptual framework. Modell (2009, p 213) observes that “abduction does not move directly from empirical observations to theoretical inferences, as is the case in purely inductive research, but relies heavily on theories as mediators for deriving explanations”.

*Retroduction:* From a description and analysis of concrete phenomena to reconstruct the basic conditions for these phenomena to be what they are. By way of thought operations and counterfactual thinking to argue toward transfactual conditions.

Critical realist philosophers have been both critical and accepting of Inductive and Deductive forms of inference (Downward et al. 2002; Downward and Mearman 2007), but argue for the added use of abstract forms of reasoning such as abduction and retroduction to the process of theory building (Danermark et al. 2002).

### Explanatory theory building method

We propose to use in this study an explanatory mode of theory building based on critical realist philosophy and methodology. There are two dominant approaches to theory building. They are:

1. *Emergent theory building* which uses predominantly inductive forms of reasoning moving from empirical observation and inquiry toward the development of theoretical concepts. In this approach the researcher enters the research situation with no *a priori* theory and allows the theory to evolve from the data. Emergent and inductive theory building has a long tradition particularly in anthropology, observational epidemiology, and the natural sciences. The approach utilises both quantitative (Tukey 1980; Hill 1965) and qualitative (Glaser and Strauss 1967; Miles and Huberman 1994) forms of empirical data and is the predominant approach to theory building used in mixed method research.
2. *Confirmatory theory testing* which uses predominantly hypothetico-deductive forms of reasoning moving from a theoretical concept to empirical testing of hypotheses. In this approach the researcher enters the research situation with an *a priori* theory and the purpose of the data collection is to “confirm” or “disconfirm” that theory. This approach has a more recent tradition (Popper 1959) cited by Rothman and Greenland (1998) and is the foundation for modern experimental science. The approach is not limited to quantitative data and has application to qualitative and mixed method confirmatory studies.

A criticism of both inductive and deductive forms of reasoning is that neither contributes to the development of explanatory theories. Haig (2005b, p 304) argues that “it is well known that conclusions of valid deductive arguments preserve the information and knowledge contained in their premises” and that although inductive arguments add new information they are only descriptive in nature. “The Scottish philosopher, David Hume, described a disturbing deficiency of inductivism: An inductive argument carried no logical force; instead, such an argument represented nothing more than an *assumption* that certain events would follow in the same pattern as they had in the past” (Rothman and Greenland 1998, p 17).

A third approach to logical reasoning embraced by critical realists is abduction and the related thought process of retroduction. This type of reasoning adds to knowledge by reasoning from “factual premises to *explanatory* conclusions” (Haig 2005b).

3. *Explanatory theory building* uses inductive, abductive, retroductive and deduction as the central forms of reasoning moving from description of the concrete, to the abstract, and back to the concrete (see Table 1) (Danermark et al. 2002, p 109–111). In this approach the researcher begins with descriptive and exploratory examination of the phenomena, events and situations intended for study. This is followed by an analytical process that involves identification of components, abduction and retroduction, comparison of theories and abstractions, and concretisation studies of the theorised mechanisms in different situations. The approach uses both quantitative and qualitative methods (Danermark et al. 2002; Danermark 2002; Haig 2005a,2005b) and is discussed further below.

**Table 1:** **The stages in an explanatory research based on critical realism (Danermark et al.** 2002 **, p 109–111)**

Stage	Description
Stage 1: Description	An explanatory social science analysis usually starts in the concrete. We describe the often complex and composite event or situation we intend to study. In this we make use of everyday concepts. An important part of this description is the interpretations of the persons involved and their way of describing the current situation. Most events should be described by qualitative as well as by quantitative methods.
Stage 2: Analytical resolution	In this phase we separate or dissolve the composite and the complex by distinguishing the various components, aspects or dimensions. The concept of scientific analysis usually alludes to just this (analysis = a separating or dissolving examination). It is never possible to study anything in all its different components. Therefore we must in practice confine ourselves to studying certain components but not others.
Stage 3: Abduction/theoretical redescription	Here we interpret and redescribe the different components/aspects from hypothetical conceptual frameworks and theories about structure and relations. This stage thus corresponds to what has been described above as abduction and redescription. The original ideas of the objects of study are developed when we place them in new contexts of ideas. Here several different theoretical interpretations and explanations can and should be presented, compared and possibly integrated with one another.
Stage 4: Retroduction	Here the different methodological strategies described above are employed. The purpose is for each one of the different components/aspects we have decided to focus on, to try to find the answers to questions like: What is fundamentally constitutive for the structures and relations(X), highlighted in stage 3? How is X possible? What properties must exist for X to be what X is? What causal mechanisms are related to X? In the concrete research process we have of course in many cases access to already established concepts supplying satisfactory answers to question of this type. In research practice, stages 3 and 4 are closely related.
Stage 5: Comparison between different theories and abstractions	In this stage on elaborates and relative explanatory power of the mechanisms and structures which have been describe by means of abduction and retroduction within the frame of stage 3 and 4. (This stage can also be described as part of stage 4.) In some cases one might conclude that one theory – unlike competitive theories – describes the necessary conditions for what is to be explained, and therefore has greater explanatory power. In other cases the theories are rather complementary, as they focus on partly different but nevertheless necessary conditions.
Stage 6: Concretization and contextualization	Concretization involves examining how different structures and mechanisms manifest themselves in concrete situations. Here one stresses the importance of studying the manner in which mechanisms interact with other mechanisms at different levels, under specific conditions. The aim of these studies is twofold: first, to interpret the meanings of these mechanisms as they come into view in a certain context; second, to contribute to explanations of concrete events and processes. In these explanations it is essential to distinguish between the more structural conditions and the accidental circumstances. This stage of the research process is of particular importance in applied science.

Both Emergent and Confirmatory modes of theory building remain supported by the *Explanatory Theory Building* approach. The *Explanatory Theory Building* approach uses deductive logic and confirmatory approaches. Danermark et al. (2002) argue that “deductive logic can and should be used in analyses of all scientific argument, regardless of what methodology is behind the results presented”. The concretisation studies, proposed by Danermark et al. (2002), are effectively confirmatory studies in different concrete situations which then contribute to the explanatory theory. Similarly Haig (2005a, p 372) argues that the hypothetico-deductive method “can play a legitimate role in hypothesis and theory testing [and] should thus be seen as complementary to the broader [abductive] theory of method, not a rival to it.”

Based on the above we have incorporated here the emergent and confirmatory theory building approaches within an overarching critical realist explanatory theory building framework.

**Figure 1:**
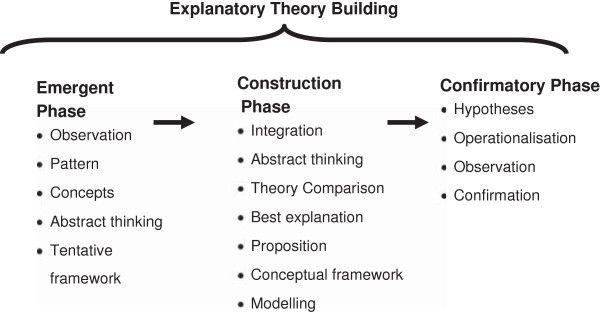
**Explanatory theory building method.**

As illustrated in Figure 1 the *Emergent Phase* leads to the development of a tentative conceptual framework. The *Emergent Phase* as described here includes mixed method inductive, deductive and abductive methods of theory generation such as exploratory data analysis, interviews, conceptual and category coding, situational analysis, exploratory factor analysis and constant comparative analysis (i.e. grounded theory). The *Construction Phase* (described as theory development and theory appraisal by Haig (2005a)) builds a conceptual framework, theory and model utilising an integration of interdisciplinary (mixed method) research, abstract thinking, comparison of theories, and identification of the best explanation(s). The *Confirmatory Phase* builds on the Concretization and Contextualisation stage described by Danermark and colleagues. Hypotheses are developed from the theoretical propositions, operationalised, and studied in concrete situations. The Confirmatory Phase of Explanatory Theory Building will not be part of the Study Protocol.

## Methods/design

### Overview

A concurrent triangulated mixed method study design will be used (Tashakkori and Teddlie 2003, p 229) for the *Emergent* and *Construction* Phases of Explanatory Theory Building. The quantitative methods will be used to detect, explore and describe phenomena; and to generate and assess theory. The qualitative methods will have an initial emergent theory orientation with preconceived ideas set aside and concepts, relationships and theory allowed to emerge from the rich qualitative data.

The setting for the study described in this Protocol is the Local Government Areas (LGA) of Bankstown, Fairfield, Liverpool, Campbelltown, Camden, Wollondilly and Wingecarribee in South Western Sydney, Australia. South Western Sydney is an area of substantial social disadvantage, and has lower education attainment and lower income levels then other parts of NSW. Based on composite socio-economic indices, about two-thirds of the area is substantially disadvantaged, which is associated with a range of poor health indicators. Ethics approval to conduct this research has been sought and obtained from the UNSW Human Research Ethics Committee.

### Critical realist study design

The Study will use a critical realist extensive-intensive study design (Sayer 2000; Jeppesen 2005). Sayer (1992) emphasised the importance of different methods of data collection and analysis. He proposes four types of research: intensive or concrete (empirical and theoretical analysis); generalisation (empirical), abstract (theoretical) and synthesis (interdisciplinary analysis). Sayer (2000) further outlines two different kinds of research design relevant to this study. The “intensive research design” is used in research where we wish to obtain in-depth knowledge of a specific phenomena for the purpose of causal explanation. “Intensive research design” mainly applies to qualitative methods. We will use here emergent theory, and the *symbolic interactionism* grounded theory method of situational analysis (Clarke 2005). “Extensive research” typically uses more quantitative methods that seek to identify regularities and patterns. The “extensive” study typically identifies regularities, or demi-regularities (Lawson 1994,1997) and has limited explanatory power (i.e. of how and why).

The role of critical realist quantitative data and statistical modelling has been controversial and requires further comment here. Mingers (2006) argues that from a critical realist perspective descriptive statistics is unobjectionable and that if patterns exist within a set of observations then there must be some underlying structures, mechanisms, or constraints that may prove to be a useful starting point for critical realist investigation. The central debate with respect to the use of inferential statistics is that they are usually used from an empiricist viewpoint where the focus is on the data itself rather than the underlying structures and mechanisms “with no attempt at explanation at all” (Mingers 2006, p 206). Mingers supports the view of Haig (2005b) that modelling techniques such as factor analysis and path analysis can go “beneath the surface to draw out latent variables and causal connections” to develop potential explanations as part of *theory generation* as described below. Mingers also describes the use of statistical methods in the assessment of competing explanations as is undertaken in the *Theory Construction* phase of this study.

Jeppesen (2005) identified the requirement to sometimes supplement the “intensive” and “extensive” designs described by Sayer (2000), with a third “explorative design” aimed at establishing an understanding of the area of investigation according to involved parties. In the study described here we will use both “intensive” and “extensive” study designs with both “exploratory” and “explanatory” phases of analysis.

### Concurrent triangulated mixed method design

**Figure 2:**
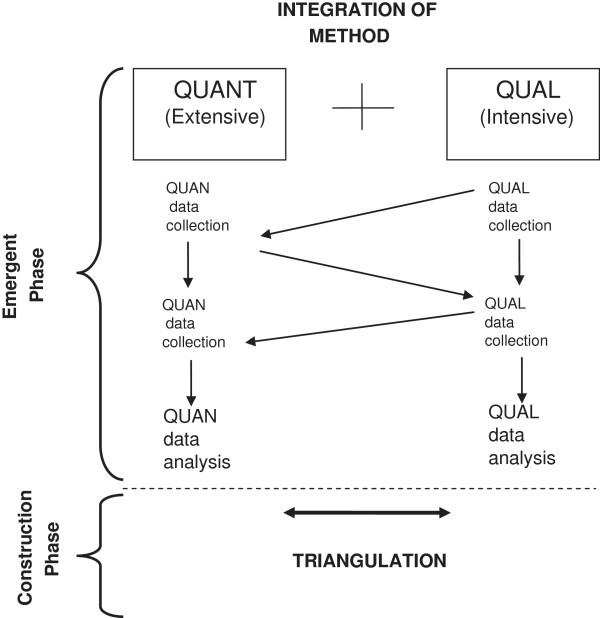
**Concurrent mixed method design.**

The concurrent triangulation design used in this Study is one of the most commonly used mixed method designs (Tashakkori and Teddlie 2003, p 229). During the *Emergent Phase* a comparative approach between qualitative (intensive) and quantitative (extensive) study arms will be used. The emerging findings will inform further exploratory analysis. The *Construction Phase* will build on the integrated analysis from the *Emergent Phase* using *triangulation,* and other theory construction methods as described in Figure 2.

### Integration

Integration of methods, data collection and analysis is the hallmark of mixed methods research (Yin 2006; Woolley 2009; Creswell et al. 2004). Yin (2006) argues that without such integration “different methods may sit in parallel, potentially leading to multiple studies, and not the desired ‘mixing’ of methods implicit in mixed methods research. Yin further proposes that integration should occur in relation to: research questions, units of analysis, samples for study, instrumentation and data collection methods, and analytic strategies.

The research design adopted for this study seeks to achieve the standards of integration proposed by Yin (2006) with integration occurring through use of common research questions, study design, units of analysis, samples for study and analytic strategies during both emergent and construction phases.

### Triangulation

*Triangulation* will be used in the Theory Construction Phase in its original trigonometrical sense pulling together micro and macro analysis and as the basis for the abductive and retroductive reasoning processes of theory construction.

Confusion has arisen in the literature concerning the use the term *triangulation* in relation to combining quantitative and qualitative approaches (Woolley 2009). Woolley (2009, p 8) observed that “its use … appears to have resulted in a common misconception … that mutual validation is the goal of mixed methods studies”. Kelle in an earlier critique of triangulation observed that there were three different understandings of the triangulation metaphor, “triangulation as mutual validation, triangulation as the integration of different perspectives on the investigated phenomena and triangulation in its original trigonometrical meaning” (Kelle 2001, p 1). Critical realism offers a further perspective on *triangulation* in mixed methods research (Modell 2009). Modell (2009, p 208) argues that critical realism counters many criticisms of *triangulation* “by re-conceptualising it as firmly grounded in abductive reasoning. This provides a foundation for maintaining researchers’ sensitivity to context-specific variations in meaning in efforts to derive theory-related explanations”.

### Emergent phase methods

The focus of our Emergent Phase is on “Phenomena Detection” and “Theory Generation”. The integration of these two theory building elements is necessary because several of the methods used in the Emergent Phase are suitable for both purposes.

#### *Phenomena detection and description*

Stage one of the critical realist approach to explanatory research described by Danermark and colleagues (Danermark et al. 2002) involves the description of the “concrete”. Haig (2005a) in his description of an “Abductive Theory of Method” also argued that the first phase is the detection and description of phenomena. For quantitative data Haig argues for the use of Exploratory Data Analysis (EDA) methods to reveal the structure or patterns in the data. Kemp and Holmwood (2003, p 178), in their paper Realism, Regulatory and Social Explanation, observe that “quantitative and statistical techniques may be used to reveal patterns…The existence of such a pattern suggests that there may be a structural influence at work”.

In relation to qualitative data Haig suggest that “Glaser and Strauss's general plea for checking the data should be taken by grounded theorists as a reminder that they should seek to reliably establish phenomena in multiply-determined ways before they begin to generate grounded theory” (Haig 1995). Thus for the grounded/emergent theorist “the data are intended to cast a wide net, as one seeks to describe, understand, and [subsequently] explain the phenomena” (Jaccard and Jacoby 2010, p 261).

Thus in the *Emergent Phase* multiple mixed methods are used including: interviews, focus groups, exploratory data analysis, exploratory factor analysis, regression, and exploratory spatial data analysis to describe the situation under study and to detect phenomena. In the multilevel study described here quantitative and qualitative methods are used at both the individual and group level to detect and describe phenomena.

#### *Theory generation*

The emergent phase of theory building moves from empirical observation toward the development of theoretical concepts. The mode of reasoning is not only inductive but also involves deductive and abductive analysis. The conceptual and categorical coding of data requires abstract reasoning and the constant comparative method (Glaser and Strauss 1967) uses both inductive and deductive reasoning in a manner that some methodologists equate as abduction. Three methods proposed by Haig (2005a) for the Theory Generation stage of theory building are: Grounded theory method, Exploratory Factor Analysis and Heuristics. Haig (2005a) observed that Grounded Theory Method and Exploratory Factor Analysis can be used for both phenomena detection and theory generation.

Analytical resolution is Stage 2 of the explanatory research approach proposed by Danermark and colleagues (Table 1). It is similar to the coding of concepts and categories in Grounded Theory Method.

“*In this phase we separate or dissolve the composite and the complex by distinguishing the various components, aspects or dimensions. The concept of scientific analysis usually alludes to just this (analysis = a separating or dissolving examination). It is never possible to study anything in all its different components. Therefore we must in practice confine ourselves to studying certain components but not others.”* (Danermark et al. 2002, p 110)

Abduction and retroduction are the third and fourth stages in the approach proposed by Danermark and colleagues (Table 1). These forms of reasoning are used in the *Theory Generation Stage* to develop tentative conceptual frameworks in both the qualitative and quantitative arms of the study. This analysis is undertaken using: categorical principal component analysis, exploratory factor analysis, exploratory confirmatory factor analysis, coding of concepts and categories, constant comparative analysis, drawing of conceptual networks, and situational analysis, to move from the “concrete to the abstract” (Danermark et al. 2002, p 109).

### Construction phase methods

The purpose of the *Theory Construction Phase* is to undertake abductive triangulation of the findings from the mixed method studies conducted in the *Emergent Phase* in order to construct a conceptual framework, theory and model. The methods used in the *Theory Construction Phase* include: 1) defining stratified levels; 2) analytic resolution; 3) abductive reasoning; 4) comparative analysis (triangulation); 5) retroduction; 6) postulate and proposition development; 7) comparison and assessment of theories; and 8) conceptual frameworks and model development.

#### *Stratified levels*

A hallmark of critical realist analysis is the ontological assumption that reality consists of hierarchically ordered levels where a lower level creates the conditions for a higher level. The higher level is not, however, determined by the lower level and has its own “generative mechanisms”. The existence of these level specific generative mechanisms is what constitutes or defines a level. The implication of this stratification is that it is not possible to reduce the causes of what occurs on one level to those on another level (whether lower or higher).

The above approach is useful as an analytical framework but “in reality levels are entwined and [the] mechanisms could be supporting each other or counteracting each other, and the outcome in a specific context is the result of a very complex interplay between levels and mechanisms” (Danermark and Gellerstedt 2004, p 350). If research focuses on one level (or two as in this study) this approach ensures that there is awareness of the existence and importance of other levels influencing the phenomena and thus is sensitive to context. In this study we will focus the analysis on mechanisms operating at the psychosocial and social levels while maintaining awareness of the existence and importance of the other levels.

#### *Analytic resolution*

Analytic resolution is undertaken in both the *Emergent* qualitative and quantitative studies as part of procedures such as coding of categories, situational analysis and factor analysis. Further analytic resolution will also undertaken during the *Theory Construction Phase*. The process contributes to the identification of the best theoretical explanation. A risk of this process is the loss of detail regarding the complexity of the processes under study. To partially address this, the analytic process involves checking back to the empirical findings in both the qualitative and quantitative studies.

#### *Abductive reasoning*

Abductive reasoning is the hallmark of realist reasoning. It is the reinterpretation and recontextualisation of individual phenomena within a conceptual framework or a set of ideas. It is about being able to understand something in a new way by observing and interpreting this something in a new conceptual framework. Modell (2009, p 213) observes that “abduction does not move directly from empirical observations to theoretical inferences, as is the case in purely inductive research, but relies heavily on theories as mediators for deriving explanations”. Peirce (1960, p 117) described the formal logic of abductive reasoning as follows:

- A surprising fact, C, is observed
- But if A were true, C would be a matter of course.
- Hence, there is reason to expect that A is true.

Eco’s (1984) typology of abduction includes overcoded, undercoded and creative types of abduction. According to Eco *overcoded abduction* is a mode of inference consisting of spontaneous interpretations based on culturally and socially prejudging. By contrast *undercoded abduction* is where we choose between a number of possible frames of interpretation or theories. Here we interpret particular phenomena as part of general theoretical structures. The third type of abduction, *creative abduction*, is characterised by being unique and innovative and moving to a frame of interpretation that nobody has used before, or which “at least opposes conventional interpretations.

We will approach the abductive process in three stages. First we will recontexualise or redescribe the phenomena identified within one of the more abstract concepts emerging from the *Emergent Phase.* This abductive inference was also imbedded within the theory generation processes associated with the empirical studies in the *Emergent Phase*. The second stage recontexualises the observed phenomena through the lens of theories arising from literature, key informants and the earlier theory generation. Finally abduction will be undertaken as part of the *Comparison Between Theories,* as discussed below.

#### *Comparative analysis (triangulation)*

We will use an integration of methods, data collection and analysis as proposed by Yin (2006) and Woolley (2009). As discussed earlier comparative analysis will also be used during the *Emergent Phase* and in this way the two arms of the study will remain integrated. In the *Theory Construction Phase* findings from the intensive (case-orientated) and extensive (variable-orientated) study designs are compared. The intensive qualitative studies provide causal explanations of possible mechanisms while the extensive quantitative studies assist with distinguishing regularities, patterns and features of the population groups. During this phase of comparative analysis the relevant literature is reviewed in more depth and treated as a third source of information for the comparative analysis.

Attention will be paid to convergence and divergence of findings. Divergence of findings will be given particular attention as it is here that “new” knowledge or understanding may be elicited through the abductive and retroductive reasoning. Thus the comparative analysis will use both abductive and retroductive processes as described here. The comparative analysis we will use is also known as “triangulation” and, as discussed earlier, will be used in its original trigonometrical sense, pulling together intensive and extensive findings and micro and macro analysis as the basis for abductive and retroductive search for generative mechanisms.

#### *Retroduction*

Retroduction is a process where we move from a description and analysis of concrete phenomena to reconstruct the basic conditions for these phenomena to be what they are. In this way thought operations and counterfactual thinking are used to argue toward counterfactual and transfactual conditions. Fleetwood observed that “with counterfactuals, the antecedents need not by instantiated; with transfactuals the consequents need not be realised” (Fleetwood 2010, p 10).

In this analysis we will look for the necessary conditions to make the phenomena possible. As discussed earlier, critical realist analysis of causal inference views structures, or entities, as having causal powers or mechanisms that have a tendency to produce events or outcomes. Contextual conditions play an important role in the realist understanding of causality because causal powers may only result in an event occurring under certain conditions. Thus outcome patterns are also contingent on context (Pawson 2006).

The methods for determining what structures and mechanisms make up the conditions for the phenomena to be possible are unclear. Danermark et al. (2002) identify six strategies which might be used. They are: counterfactual [and transfactual] thinking, thought experiments, social experiments, studying pathological circumstances and extreme cases, and comparison of different cases.

#### *Postulate and proposition development*

Theoretical statements can be expressed as postulates, propositions or hypotheses. There is a logical order in which these terms are used here moving from the general to the specific. In this phase we will focus on the development of proposition statements leaving hypothesis generation till later. A proposition is defined by Hellevik (1984) as a statement about the relationship between variables. From a hypothetico-deductive perspective Dubin (1969, p 205) states that propositions are often expressed as “if *a* then *b*” deductive statements. Dubin (1969, p 205) further observes that:

*“A proposition is a truth statement about a model when the model is fully specified in its units, laws of interaction, boundary, and system states. Any truth statement that can be made about such as system is a proposition of the system”*

The hypothetico-deductive approach to theory building requires a “closed” system. To achieve this Dubin argues that boundaries and system states must be defined. The approach also requires that the theory be tested within the empirical world with “things observable” (Dubin 1969, p205).

By contrast critical realism views reality as an “open system” where causative processes are always contextually determined. Smith (2008, p 5) states that critical realist explanation includes:

*“the structure that underlies the generative mechanisms (structure of X), the outcome that these mechanisms tend to produce (Y), and finally the elements of context that trigger or inhibit the firing of these generative mechanism (C). Any explanation must include all three of these elements”*

**Figure 3:**
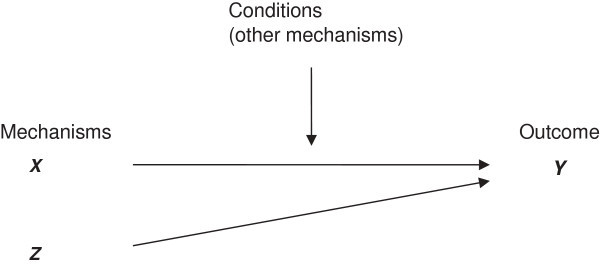
**Graphical representation of critical realist propositions (Danermark** 2002 **).**

Thus realist theoretical propositions are about how “mechanisms (M) are fired in contexts (C) to produce outcomes (O)” (Pawson and Tiley 1997, p 85). Explanation cannot begin without the identification of the generative mechanism and their underlying structures. Causal relationships only occur when the generative mechanism comes into operation. Sometimes different mechanisms produce the same outcome. The contextual conditions determine whether the generative mechanism(s) will come into play and the nature of the outcome. The contextual conditions include other mechanisms that may either trigger or counteract the causal mechanism. This can be illustrated as in Figure 3.

The graphical representation of a critical realist proposition (Figure 3) will be used in our study to summarise the findings of the comparative analysis (triangulation), and abductive and retroductive reasoning.

#### *Comparison and assessment of theories*

Critical realist methodologist Danermark et al. (2002), and Haig (2005a) both identify a stage in explanatory research and theory construction where comparison and assessment of the identified theories and abstractions is undertaken. The purpose here is not to test or confirm the theoretical propositions but use further abductive reasoning to identify the *Best Explanation*.

Danermark et al. (2002) suggest that in this stage one elaborates and estimates the relative explanatory power of the mechanisms and structures identified by previous abduction and retroduction. One theory is then said to have more explanatory power than another theory about the same subject matter if it can predict and otherwise account for all the facts that the second one does, but also explains the causes of other facts which the second one does not.

Haig (2005a) proposes the use of “inference to the best explanation, which accepts a theory when it is judged to provide a better explanation of the evidence than its rivals do”. Haig further argues for the use of Thagard’s (1992) formulation of inference to the best explanation, which identifies, and systematically uses, a number of evaluative principles and criteria in a way that has been shown to produce reliable judgments of best explanation in science. Thagard’s seven principles are: symmetry, explanation, analogy, data priority, contradiction, competition, and acceptability. The determination of the explanatory coherence of a theory is made in terms of three criteria: consilience, simplicity, and analogy (Thagard 1988).

Ward (2009) recently critically reviewed the role of Hill’s “criteria” and argued that they are not appropriate for either deductive or inductive inferences but that they have an important role to play in abductive inferences to the best explanation. This conclusion clearly places the commonly used Bradford Hill “criteria” within a realist epistemology and therefore appropriate for assessment of the theories constructed in this study.

Inference to Best Explanation is thus an abductive mode of inference considered by some philosophers of science to be an alternative to the hypothetico-deductive and Bayesian probabilistic approaches to evaluation of theory (Thagard 1978). We propose the use of both the Thagard and Hill principles of best explanation as part of the abductive process for social epidemiology.

#### *Conceptual frameworks and models*

The terms “conceptual frameworks”, “theories” and “models” are often used in interchangeably and together. We will use the typology proposed by Carpiano and Daley (2006) where the different levels of abstraction move from the broadest level of conceptualisation (framework) to the more focused (model). Many grounded theorists treat creating visual images of theory as an intrinsic part of grounded theory methods (Charmaz 2006, p 117). Such methods are consistent with critical realist qualitative and quantitative methodology. Miles and Huberman (1994, p 222-238) also give an extensive account of the use of causal chains, networks and models including the use of factor analysis and modelling of variable relationships.

A *model* is the narrowest in focus and is used to make specific assumptions about a limited set of parameters and variables. A model may draw on several theories and when presented as a diagram a conceptual model may provide a visualisation of proposed causal linkages (Carpiano and Daley 2006).

Borsboom et al. (2003) have examined the theoretical status of latent variables as used in theoretical models and argued that such models require a realist ontology as presented here. Using the *Latent Variable Theory* proposed by Borsboom (2008) we argue that social level variables, as studied in social epidemiology, should also be considered latent (i.e. representations of unobserved structures and mechanisms) until proven as observed. We will therefore use models to visually represent proposed: (a) causal flow, (b) structures, mechanisms and outcomes, and (c) causal connections between variables (observed or latent).

## Discussion

We have described here a study protocol that will use the meta-theory of critical realism for the generation of causal explanations in social epidemiology as a response to the criticisms put forward by Muntaner (1999), O'Campo (2003) and Raphael (2006). The development of realist methodologies in epidemiology and population health is relatively new (O'Campo and Dunn 2012). The study will demonstrate that critical realism can provide the necessary meta-theoretical philosophy for the generation of social epidemiology theory. By stratifying reality critical realism demands that the researcher examines and explains the unobserved generative forces (i.e. social exclusion) that shape the experiences of their human subjects. The fallibility of observations (and thus knowledge) is partly explained by the ontological separation of actual and observed realms, together with the influence of context on the generative mechanism(s) and experienced phenomena.

As a meta-theory critical realism seems to be ideally suited for social epidemiology theory building and testing. Qualitative methods for confirmatory studies are well supported by critical realism (Sayer 2000; Danermark et al. 2002) and realist approaches are gaining credibility in relation to evidence-based policy and programme evaluation (Pawson 2006; O'Campo et al. 2009a; McGuire 2005). From a critical realist perspective quantitative modelling can be very useful to test out possible explanations (Mingers 2006) but findings are “not assumed as closure on reality” (Olsen and Morgan 2005). The “demi-regularities” identified, and hypotheses refuted, do not necessarily reveal “laws” and scepticism is required.

We have identified two dominant approaches to theory building, namely: emergent theory building and confirmatory theory testing. Explanatory theory building has been described by Haig (2005a) and Danermark et al. (2002) and we incorporated here the emergent and confirmatory theory building approaches within an overarching critical realist explanatory theory building framework. The resulting pluralistic and transdisciplinary *Explanatory Theory Building Method* has the potential to make a significant contribution to population health and social epidemiology theory building.

The *Emergent Phase* draws on strong qualitative emergent and grounded theory and quantitative exploratory data analysis traditions with their strength for theory generation. The *Construction Phase* makes explicit the abstract analytical process of abduction and *Inference to Best Explanation (IBE)* from where hypothetico-deductive theory testing typically starts and emergent theory building finishes (Lynham 2002). The *Confirmatory Phase* embraces case study, probabilistic and hypothetico-deductive methods within a realist philosophy where propositions are examined in concrete situations, “demi-regularities” identified, hypotheses refuted or confirmed, but always with scepticism of establishing “laws” that will be later found fallible.

The study will also demonstrate that the emergent and construction phases of *Explanatory Theory Building Method* can be applied to the field of social epidemiology and population health theory building as they have in disability (Danermark and Gellerstedt 2004) and development research (Olsen 2001). Confirmatory approaches within a realist philosophy have also been successfully demonstrated within social epidemiology (O'Campo et al. 2009a) and health policy and programme evaluation (Greenhalgh et al. 2009).

The strength of the critical realist approach is the extent to which this paradigm can support the epistemological, ontological, axiological, methodological and rhetorical positions of quantitative and qualitative research in the field of social epidemiology.
